# Skin Lesion Area Segmentation Using Attention Squeeze U-Net for Embedded Devices

**DOI:** 10.1007/s10278-022-00634-7

**Published:** 2022-05-03

**Authors:** Andrea Pennisi, Domenico D. Bloisi, Vincenzo Suriani, Daniele Nardi, Antonio Facchiano, Anna Rita Giampetruzzi

**Affiliations:** 1 Dept. of Computer Science, University of Antwerp, Antwerpen, Belgium; 2 Dept. of Mathematics, Computer Science, and Economics, University of Basilicata, Potenza, Italy; 3 Dept. of Computer Science, Control, and Management Engineering, Sapienza University of Rome, Rome, Italy; 4 Istituto Dermopatico dell’Immacolata IDI-IRCCS, Rome, Italy

**Keywords:** Melanoma detection, Image segmentation, Deep learning

## Abstract

Melanoma is the deadliest form of skin cancer. Early diagnosis of malignant lesions is crucial for reducing mortality. The use of deep learning techniques on dermoscopic images can help in keeping track of the change over time in the appearance of the lesion, which is an important factor for detecting malignant lesions. In this paper, we present a deep learning architecture called Attention Squeeze U-Net for skin lesion area segmentation specifically designed for embedded devices. The main goal is to increase the patient empowerment through the adoption of deep learning algorithms that can run locally on smartphones or low cost embedded devices. This can be the basis to (1) create a history of the lesion, (2) reduce patient visits to the hospital, and (3) protect the privacy of the users. Quantitative results on publicly available data demonstrate that it is possible to achieve good segmentation results even with a compact model.

## Introduction

Melanoma is an extremely aggressive and lethal skin tumor. It takes one life in every 54 min in the USA and one person dies every 5 h from melanoma in Australia [1]. In Europe, over 100,000 new melanoma cases and 22,000 melanoma related deaths are reported annually [2]. Early detection is crucial for survival, since melanoma is capable of spreading quickly and thus needs to be treated urgently.

Dermoscopy is a non-invasive and cost-effective technique for detecting early-stage skin cancer by helping dermatologists in individuating visual lesion features that are not discernable by examination with the naked eye. Dermoscopic images are generated by combining a low angle-of-incidence lighting with optical magnification obtained using either liquid immersion or cross-polarized lighting. Structure information inferred from dermoscopic images is used to apply the ABCDE (Asymmetry, Border, Color, Diameter, Evolution) rule, which is based on the assumptions that most early melanomas are asymmetrical (A), melanomas usually present uneven borders (B), melanoma has a variety of colors while most benign pigments have one color (C), in most cases, melanomas have a diameter larger than 6 mm (D), unlike the majority of benign lesions, melanoma tends to evolve or change over time (E).

Dermoscopy has two main drawbacks:

1. It requires a specific training.
2. Even with sufficient training, visual analysis remains subjective.

To overcome the above listed limitations, a number of Computer Aided Diagnosis (CAD) systems have been proposed. In particular, deep learning (DL) based methods for dermoscopy image analysis (DIA) have the potential to improve skin cancer detection rates, since they proved to be superior to dermatologists in melanoma image classification [3]. Even though DL methods are not replacement solutions for medical doctors, melanoma screening using DL techniques is a promising solution to improve management and prognosis of skin cancer by promoting earlier diagnosis [4]. In fact, DL algorithms can potentially run on embedded systems (including smartphones) and be used to improve patients’ empowerment by directly involving the patients themselves in monitoring over time their lesions.

Local execution of skin lesion detection tools has three advantages with respect to sending images to web servers for processing [5]:

1. Storing images on the local memory of the embedded system (instead of sending them over the Internet) allows to better preserve the patient’s privacy.
2. Computational power on embedded systems has generally a much lower cost and lower power consumption than on general purpose PCs.
3. On-Device computation on embedded systems leads to low-latency applications since the device can compute and process data locally.

**Fig. 1:**
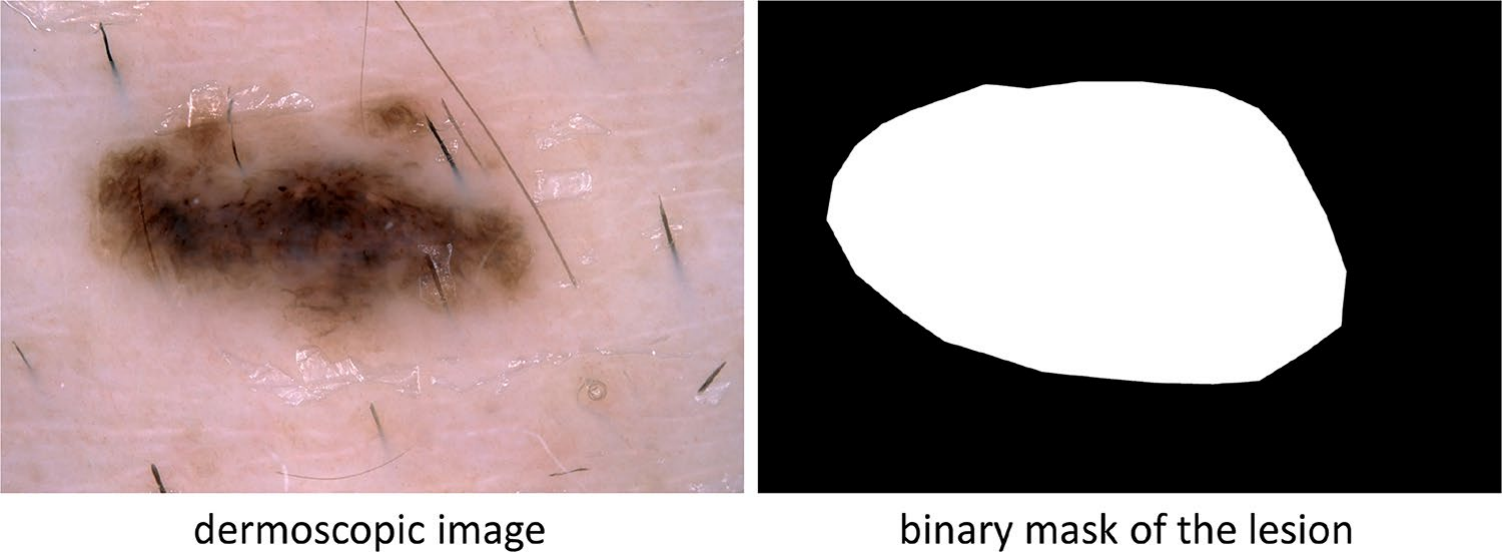
Lesion area segmentation. Left: Dermoscopic image in input. Right: Binary mask in output, where white pixels belongs to the lesion area and black pixels are extraneous to it. [Images from ISIC 2017]

**Fig. 2:**
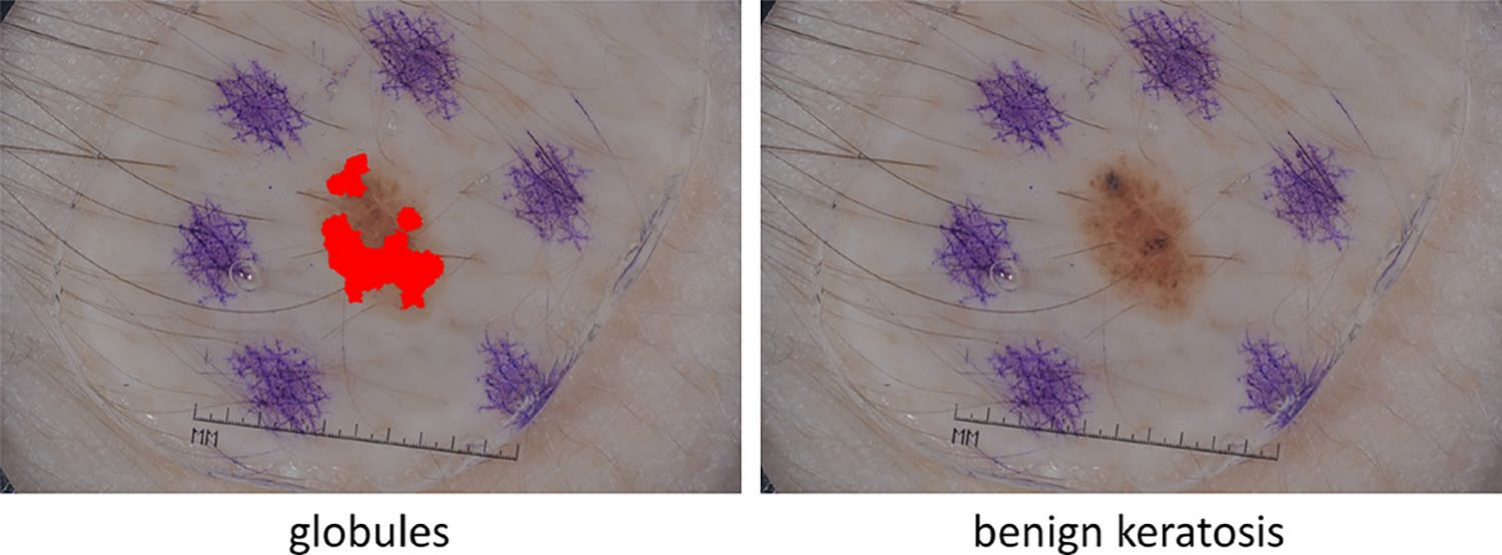
Left: The goal of attribute detection is to localize clinical dermoscopic criteria in the image in input (in this case, globules are highlighted in red). Right: Classification concerns the labelling of the image in input according to different diagnostic classes (in this case, the assigned label is “benign keratosis”). [Images from ISIC 2017]

DL methods can be applied to address three primary tasks, namely (i) lesion area segmentation, (ii) lesion attribute detection, and (iii) disease classification. The goal of the **lesion area segmentation** task is to create a binary mask from a dermoscopic image that provides an accurate separation between the lesion area and the surrounding healthy skin (see Fig. 1). **Attribute detection** aims at localizing clinical dermoscopic criteria that have been found to be correlated with disease states, such as pigment network, negative network, streaks, milia-like cysts, and globules (see the left side of Fig. 2). In **classification**, the images in input are labelled according to different diagnostic classes. Beyond the typical categorization into benign and melanoma, it is possible to group dermoscopic images into more than two classes. This provides a better discrimination between melanoma, other types of skin cancer that are less aggressive than melanoma, and benign lesions. For example, Celebi et al. in [2] propose a classification based on seven classes, including melanoma, melanocytic nevus, basal cell carcinoma, actinic keratosis, benign keratosis, dermatofibroma, and vascular lesion (see the right side of Fig. 2).

In this paper, we focus on the lesion area segmentation task using a deep convolutional pixel-wise method that can run on embedded devices. While any medical interpretation of skin lesion segmentation can be only performed by experts, a history of the lesion can be created by the patient independently at home.

The main novelty consists in combining a squeeze based approach with an attention block, thus forcing the squeeze layers to focus on the features coming from the attention block. In such a way, the network learns only the main features that represent the lesion area, without the need of introducing a huge amount of parameters to learn. This makes our network suitable to run on low cost and portable embedded devices, which could be used by the patients themselves. Moreover, as a difference with cloud-based approaches (e.g., [6]), in our approach, which can be completely performed on an embedded board, we do not need to send the images of the lesions over the Internet, thus avoiding potential risks for the patients’ privacy.

The contribution of this work is threefold. First, we describe a compact architecture for dermoscopic image segmentation, called Attention Squeeze U-Net. Second, we compare different network architectures on publicly available data using different datasets for the training and the test phases, in order to evaluate their generalization capability. Third, we provide a per-lesion class analysis of the segmentation results.

The remainder of the paper is organized as follows. The following “Related Work” contains an overview of recent methods for lesion area segmentation. “Material and Methods” presents the details of our artificial network and “Training” describes its training. Qualitative and quantitative experimental results are shown in “Experimental Results”, including a detailed discussion of the results per lesion class. Finally, conclusions are drawn in “Conclusions”.

## Related Work

**Fig. 3:**
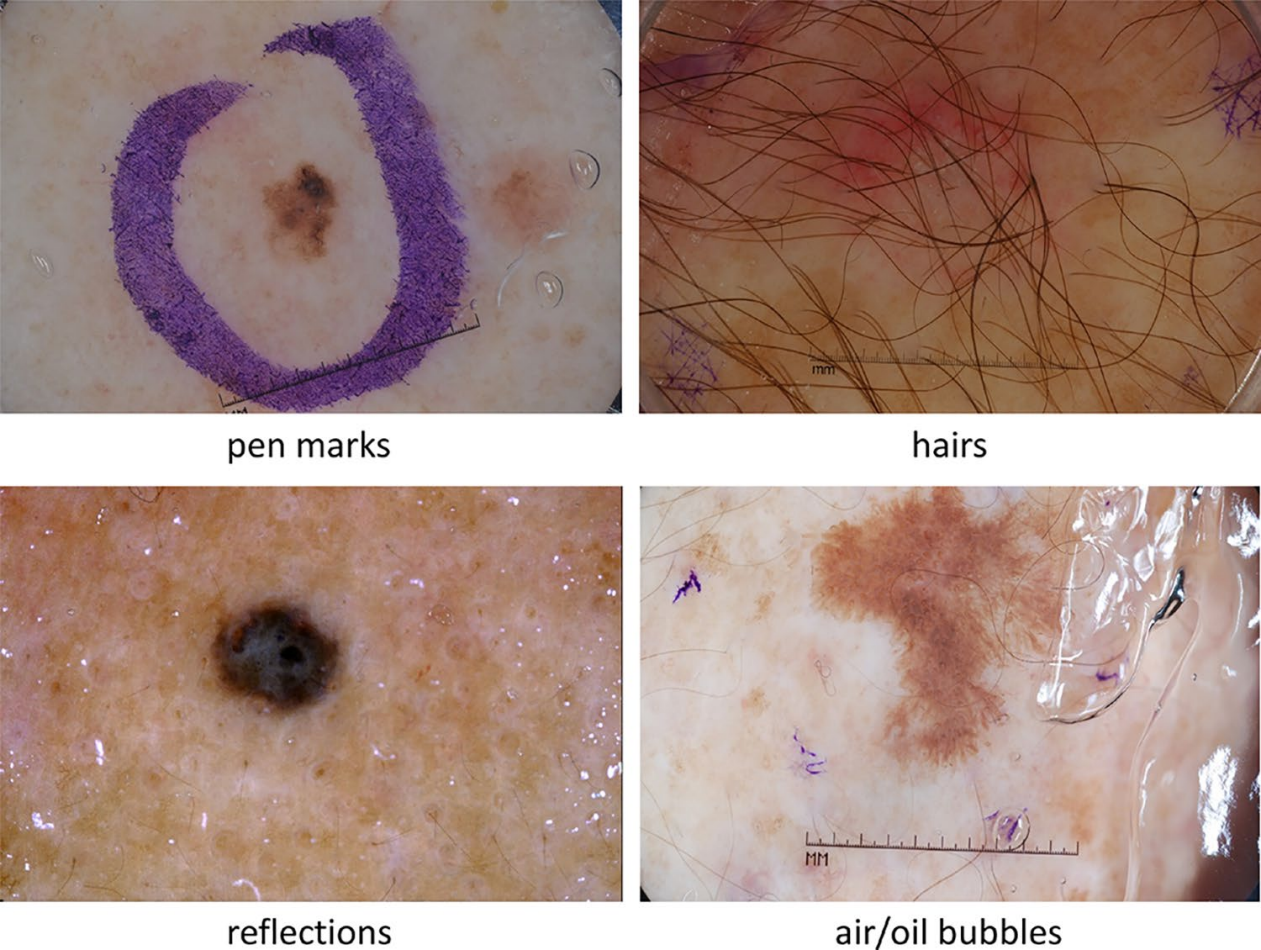
Typical artifacts in dermoscopic images. Top left: Pen marks around the lesion. Top right: Hairs over the lesion. Bottom left: Presence of specular reflections. Bottom right: Air/oil bubbles due to the use of an interface fluid. [Images from ISIC 2017]

Accurately segmenting the lesion area is extremely important for performing a temporal analysis of its visual features on a quantitative basis. In fact, a melanocytic naevus usually does not change its size, shape, and color, whereas the visual appearance of a melanoma can change over time. When lesion area segmentation methods are used on dermoscopic images, the main challenges to deal with are (see Fig. 3):

- The multiple lesion shapes, size, colors, skin types, textures, and the eventual presence of artifacts.
- The limitations of large and annotated publicly available databases, which are small, heavily imbalanced, and contain images with occlusions [1].

Early work in dermoscopy image segmentation used handcrafted feature-based methods, such as thresholding, clustering, and graph partitioning, to obtain the binary mask of the lesion [7]. Despite the positive results, methods based on handcrafted features are strictly dependent on the choice of the features. This limits their generalization capabilities especially when dealing with the great variety of lesion types in input.

To overcome the inflexibility and limitations in terms of expressiveness of handcrafted vision pipelines, dermoscopy image segmentation systems moved toward an end-to-end approach based on DL methods, such as Convolutional Neural Networks (CNNs). These data-driven methods allow to train powerful visual classifiers that report high classification performance. However, their results strongly depend on the size and variety of the training dataset [8].

The problem of lack of data has been addressed by setting up collaborations between academia and industry to improve melanoma diagnosis. From 2016, the International Skin Imaging Collaboration (ISIC) organizes an annual open challenge on a public archive of clinical and dermoscopic images of skin lesions. In particular, ISIC Challenge 2017 and 2018 provided a specific task about lesion segmentation, with a considerable number of 2,594 training images (plus corresponding ground truth segmentation masks) for the 2018 challenge [9].

The first place for Task 1 - Lesion Boundary Segmentation at ISIC Challenge 2017 was achieved by a submission using a deep fully convolutional-deconvolutional neural network [10] with 29 layers [11]. The 2017 second ranked submission used U-Net [12] with input images resized down to 192$\times$192 pixels [13]. ResNet [14] was used by the third placed submission [15]. In 2018, the winning submission used a two-stage pipeline [16]. The first step was a detection process based on MaskRCNN to find a bounding box of the lesion in each of the input images in order to crop them. In the second step, the cropped images were segmented using an encoder-decoder architecture based on DeepLab and PSPNet. The 2018 second placed submission [17] also was based on the DeepLab model with a transfer learning taking pre-trained weight on VOC PASCAL 2012. The third place went to a U-Net based model [18], where information about low level features is preserved thanks to the addition of multiplications between feature maps before each connection in the encoder part of the net.

The web page https://paperswithcode.com/sota/lesion-segmentation-on-isic-2018 contains a list of papers about lesion segmentation on ISIC 2018 with the source code available. Among them, Bencevic et al. in [19] present a method, called *Polar Res-U-Net++*, for improving the segmentation performance by using a polar transformation to convert the images from the Cartesian space to the polar one. In such a way, the dimensionality of the image is reduced as well as the segmentation and localization tasks are separated; thus, the network is able to converge more easily. The method is very effective in segmenting liver, polyp, skin lesion, and epicardial adipose tissue. Azad et al. [20] propose a Bi-directional ConvLSTM U-Net with Densely connected convolutions (BCDU-Net), which is an extension of U-Net that integrate the bi-directional ConvLSTM (BConvLSTM) [21] with the mechanism of dense convolutions for the segmentation of medical images. BConvLSTM is used to combine the feature maps extracted from the corresponding encoding path with the previous decoding up-convolutional layer in place of using the standard U-Net skipping connections. Then, a densely connected convolution layer is used in the last convolutional layer of the encoding path for strengthening the feature propagation and encouraging the feature reuse. The approach achieves state-of-the-art results on retinal blood vessel segmentation, skin lesion segmentation, and lung nodule segmentation. Jha et al. in [22] describe an architecture called Double U-Net, which uses two U-Net architectures in sequence, with two encoders and two decoders. The first encoder is based on VGG-19 (pre-trained on ImageNet). The Atrous Spatial Pyramid Pooling (ASPP) is used to capture contextual information within the network. Such an approach has been tested on several domains (i.e., polyp segmentation, skin lesion segmentation, and nuclei segmentation) obtaining remarkable results.

In this work, we propose the use of an Attention Squeeze U-Net architecture for pixel-wise segmentation on dermoscopic images. The aim is to design and test a compact network architecture that can run on embedded devices with similar performance of larger architectures that need powerful GPUs to run. We believe that the development of robust DL segmentation methods that can run on smartphones is the first step toward the adoption of a patient-centered paradigm for the early detection of melanoma.

## Material and Methods

The proposed model for lesion area segmentation is called Attention Squeeze U-Net and it is inspired by the following architectures:

- U-Net [12]
- Squeeze U-Net [23]
- Attention U-Net [24]

### U-Net Architecture

U-Net is an encoder-decoder model developed for medical and biomedical applications. Its symmetrical architecture, which looks like a “U’’, makes it particularly suited for image segmentation for the following reasons. To solve classification problems, DL approaches create a feature map of an image and convert it into a vector, which is then used for classification. In image segmentation, DL methods also convert the feature map of an image into a vector, but also generate a mask image from that vector. Due to the loss of information in the encoding stage, converting the feature vector into an image can generate distortions. The idea in U-Net is to store information about the transformation applied at each encoding stage in order to use it in the decoding stage, thus facilitating the generation of the mask image from the feature vector, by preserving its structural integrity. However, U-Net (in its Keras implementation) has more than 30 million trainable parameters, a considerable number when dealing with the limited computational power and memory of an embedded device. The need of computing millions of parameters slows down the inference process and can lead to errors related to exhausted resources.

### Squeeze U-Net Architecture

**Fig. 4:**
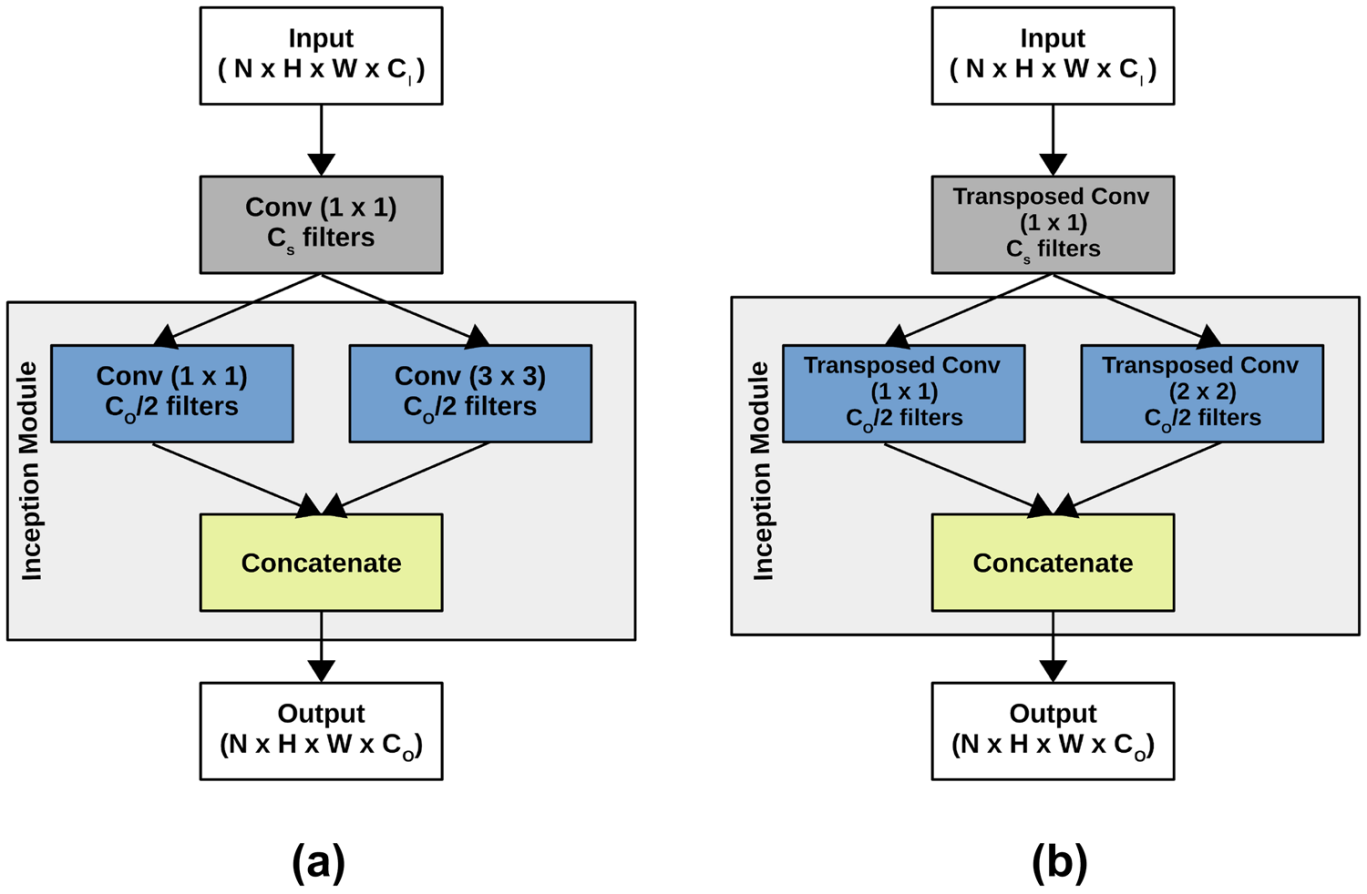
Fire Blocks. **a** Convolutional fire block in the contraction path. **b** Transposed convolutional fire block in the expansion path

Modifications of U-Net have been proposed to reduce the model size. In particular, Squeeze U-Net [23] is a memory and energy efficient model inspired by U-Net, where the down- and upsampling layers are replaced by *fire* modules. Figure 4 shows the structures of the so-called fire blocks. A fire module, introduced in SqueezeNet [25], uses fire point-wise convolutions together with an inception stage [26], which are then concatenated to form the output. In such a way, the Squeeze U-Net model needs only 2.5 millions parameters, more than ten times less than U-Net. In the contraction path, each fire module (see Fig. 4a) is made of a 1$\times$1 convolutional layer with $C_{S}$ (squeeze) channels followed by an inception block with 2 convolutions of 3$\times$3 and 1$\times$1, respectively, with $C_{O} / 2$ channels. The resulting $C_{O}$ channels are concatenated in order to get the desired output, which is then passed to the next layer and to the skip connection of the network.

In the expansive path, the main component is the upsampling block (see Fig. 4b). In each block, the transposed fire module is made of a 1$\times$1 transposed convolutional layer, followed by an inception block consisting of 2 parallel 1$\times$1 and 2$\times$2 convolutional layers that are concatenated for obtaining the output. The upsampling blocks are then used with the skip connection in order to merge the high resolution features of the contraction path with the low resolution features of the expansive path.

### Attention U-Net Architecture

**Fig. 5:**
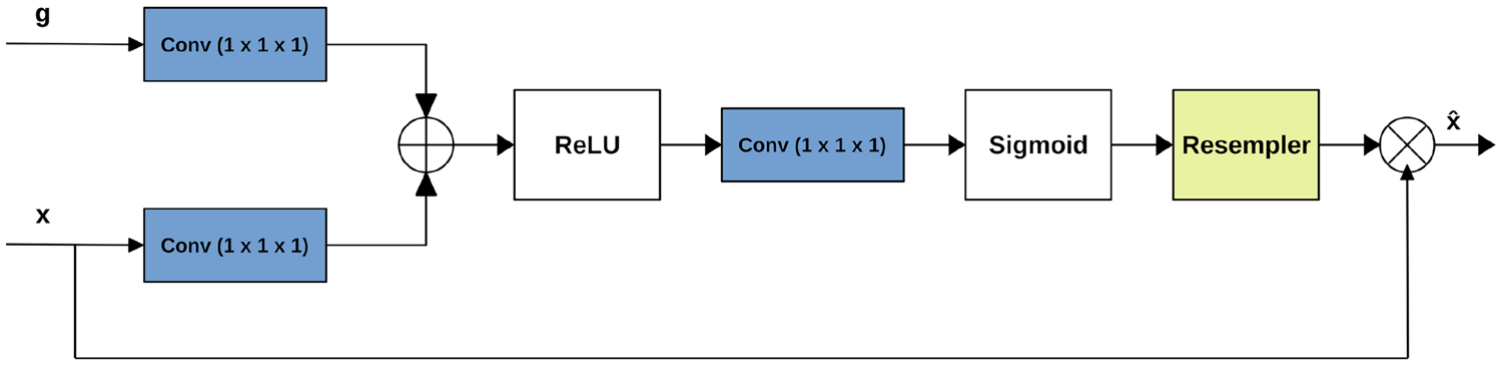
Attention block

Squeeze U-Net is successful in reducing the number of parameters to learn from the 30 million in U-Net to only 2.5 million. However, the concatenation mechanism in Squeeze U-Net can be a limiting factor when dealing with medical images, since all the high level features are concatenated with all the low level features with the risk of losing many useful information. For solving this problem, it possible to introduce an attention block (see Fig. 5) into the upsampling block [24]. In particular, the attention mechanism is integrated into the skip connections. An attention block takes two inputs: *g*, coming from the previous block, and *x*, coming from the skip connection. It is worth noticing that *g* has smaller size (but better feature representation) than *x*, thus it needs to be processed by an upsampling layer before the attention block in order to achieve the same size of *x*. Both *x* and *g* are fed into 1$\times$1 convolutions, in order to have the same number of channels without changing the size of the layers. Then, *g* and *x* are summed and the resultant vector goes through a ReLU activation layer and a 1$\times$1 convolution that collapses the dimensions to a 1$\times$H$\times$W vector. This last vector is given to a sigmoid layer, which scales the vector in the range [0, 1], thus producing an attention map (weights), where each value close to 1 indicates a relevant feature. Finally, the attention map is multiplied by the skip input to produce the final output of the attention block.

As stated above, the idea behind U-Net is to let the features from the contraction path guide the features of the expansion path by concatenating them. Applying an attention block before the concatenation allows the network to understand which features from the skip connection are more relevant and to weight them more. Thus, by multiplying the skip connection and the attention distribution, the network can focus on a particular part of the input, rather than feeding in every feature.

### Attention Squeeze U-Net Architecture

We propose here a novel network called Attention Squeeze U-Net, which contains a special upsampling block. This upsampling block takes as input the previous output of network *g* and the skip connection *x*. A transposed convolutional operation is applied to *g* in order to obtain $\hat{g}$, which is sent as input to an attention block together with *x*. The output of the attention block is concatenated with $\hat{g}$ and given as input to a fire block. The above described modification of the upsampling block allows:

1. To maintain the model lightweight, as in Squeeze U-Net.
2. To add the attention mechanism to Squeeze U-Net obtaining better segmentation results.

**Fig. 6:**
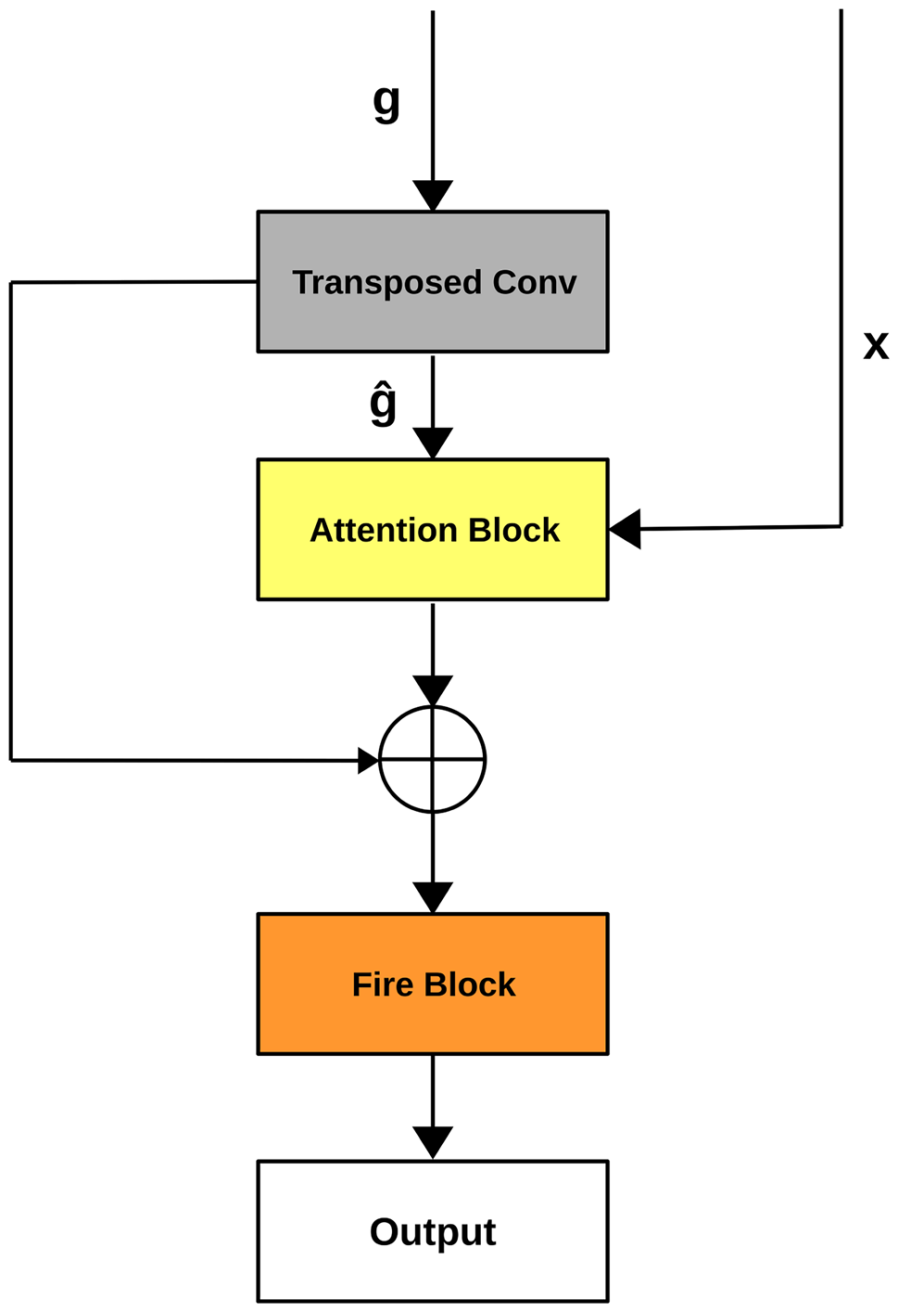
Upsampling Block

**Fig. 7:**
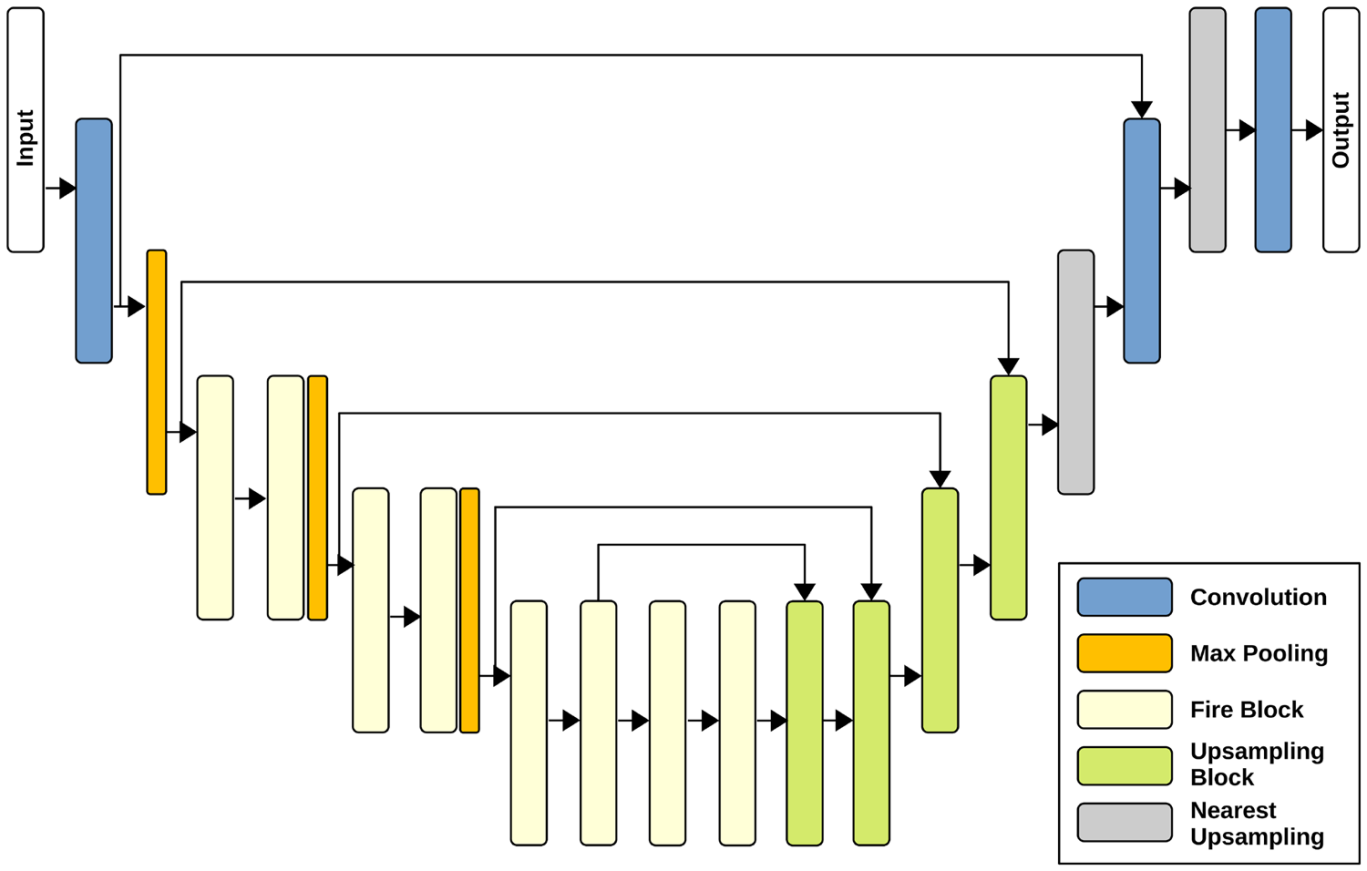
Attention Squeeze U-Net

Figure 6 shows the details of the upsampling block, while the architecture of our Attention Squeeze U-Net is shown in Fig. 7.

The contractive path of the network is made of a convolutional layer with a stride of 2 $\times$ 2, followed by a set of fire blocks and max pooling operations. The expansive path includes four upsampling blocks, two convolutional layers and two upsampling blocks based on the nearest neighbor approximation. The number of parameters in Attention Squeeze U-Net is only $\approx 100k$ more than Squeeze U-Net, thus allowing for real-time performance on embedded devices.

### Adaptations for Embedded Systems

To run on embedded systems (including smartphones), our model needs some small adaptations. Our intention is using the embedded device in the inference phase, while, for training, it can be used a more powerful GPU on a desktop PC.

To deploy Attention Squeeze U-Net on an Android phone, we use the open source framework NCNN [27], which is a high-performance neural network inference computing framework strongly optimized for mobile platforms. NCNN supports acceleration through ARM NEON vectorization and provides NEON assembly implementation for the computationally intensive convolution kernels of CNNs. To create an Android application able to make inference by using Attention Squeeze U-Net, we converted the Tensorflow 2 model in an NCNN one. The application has been developed by using Android NDK^1^ to use a native C code, and Vulkan SDK^2^ to reduce the CPU overhead.

**Fig. 8:**
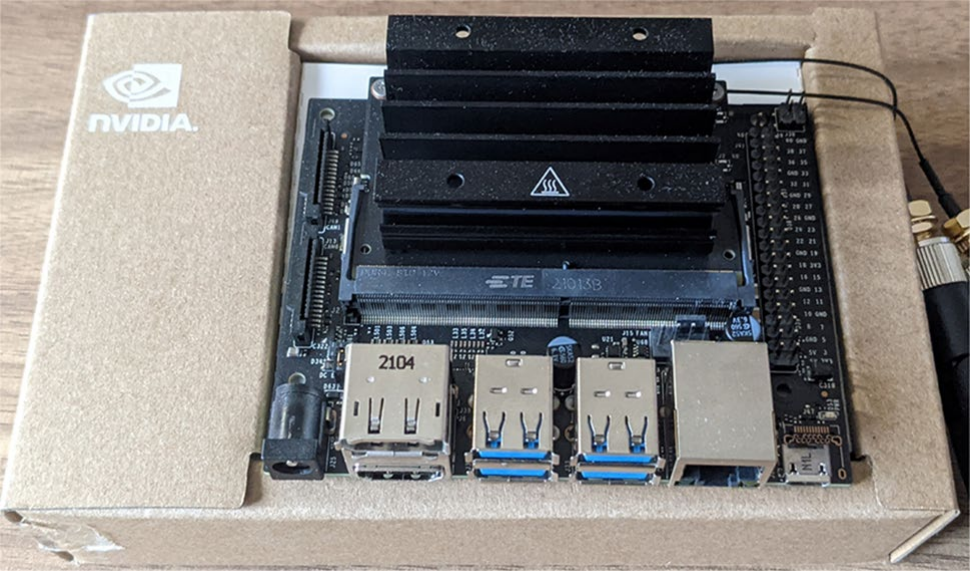
NVIDIA Jetson Nano embedded board

To run our network on an Nvidia Jetson Nano (see Fig. 8), which is a low cost (about 100 USD) embedded card commercialized by NVIDIA^3^, we do not employ any optimization. It is worth noticing that, the network can be optimized by using TensorRT^4^, a framework provided by NVIDIA.

## Training

Our training set is made of dermoscopic images and corresponding ground truth annotations coming from the ISIC 2017 dataset [28]. In particular, we use the following data from ISIC 2017 as training and validation sets:

1. All the 2,000 dermoscopic images from the training data folder in JPEG format.
2. The corresponding 2,000 binary mask images in PNG format from the training ground truth data folder.
3. All the 150 dermoscopic images from the validation data folder in JPEG format.
4. The corresponding 150 binary mask images in PNG format from the validation ground truth data folder.

The above described training and validation data have been downloaded from the following URL: https://challenge.isic-archive.com/data#2017 Since ISIC 2017 have different image sizes, all the images have been resized to 384$\times$512 pixels and then normalized between 0 and 1 before sending them to the models.

It is worth noticing that:

1. A considerable number of images contain artifacts such as air/oil bubbles, body hairs, and colored band-aids.
2. The labelling of the skin lesions does not follow a predefined pattern, since the annotations may have been done by different experts or with the help of semi-automated algorithms.

For the above listed reasons, we consider the background (i.e., the black pixels) in the ground truth masks as a class, thus treating the lesion segmentation task as a multi-class classification problem.

A data augmentation technique has been used to increase the number of the training samples. In particular, we used three transformation for each original image: vertical flipping, horizontal flipping, and both. The augmentation procedure increases the number of training samples to 8,000 images.

### Loss Function

DL image segmentation networks are usually trained using a (weighted) cross-entropy loss. However, the evaluation of the segmentation results in medical imaging is commonly based on the Dice score and the Jaccard index (see “Performance Metrics” for details) to deal with the problem of class imbalanced datasets, which is frequent in the medical domain. The use of a learning optimization objective (the so-called loss) function different from the evaluation metric used for the test data introduces an adverse discrepancy [29]. In fact, cross-entropy and its weighted version are inferior to metric-sensitive loss functions (such as soft-Dice and soft Jaccard) when evaluated on the Dice score and the Jaccard index.

In order to avoid the above discussed discrepancy between the loss function used during the training phase and the metrics considered for evaluating the results, we have decided to use the Focal Tversky loss (FTL) as the loss function to train our Attention Squeeze U-Net network. FTL [30] is a generalization of the Tversky index (described in “Performance Metrics”), which in turn generalizes the Dice coefficient and the Jaccard index.

FTL can be defined as:

1 $$\begin{aligned} FTL = \left( 1-TI\right) ^{\gamma } \end{aligned}$$

where TI is the Tversky index, while $\gamma$ is a parameter that controls the non-linearity of the loss. When $\gamma$ tends to $+\infty$, the gradient of the loss tends to $\infty$, while TI tends to 1. If $\gamma$ tends to 0, the gradient of the loss tends to 0 and TI tends to 1. Thus, when training sample presents a value for $\gamma < 1$, the gradient of the loss is higher, thus forcing the model to focus on such samples. This property is particularly useful in the final stage of the training process, since the model is encouraged to continue to learn even though TI is nearing convergence.

FTL is particularly suited in the case of datasets affected by class imbalance. In fact, when $\gamma > 1$, the model is forced to focus on “hard” samples, i.e., images with a small foreground region, where usually the TI has a low score. Moreover, the non-linear nature of FTL permits to control how the loss behaves at different values of the obtained Tversky index.

## Experimental Results

In this section, firstly we provide a description about different performance metrics with a discussion about their usage. Then, we describe the two test sets, i.e., ISIC 2017 and PH2. Finally, we show the quantitative results of the comparison between our approach and other three well-known models on the two test sets.

### Performance Metrics

We have two sets to compare:

1. The predictions set, which is made of the segmentation masks generated by the trained model.
2. The ground truth masks set, which represents our goal.

**Fig. 9:**
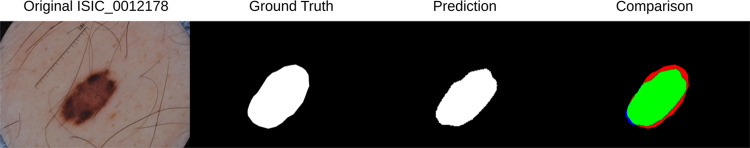
Predicted and ground truth masks are compared in terms of the number of true positive (green pixels in the comparison image), false positive (red), false negative (blue), and true negative (black) pixels. [The two leftmost images are from ISIC 2017]

By comparing the predictions set and the ground truth set, we can get a measure of how good is our model. The quantitative comparison can be carried out in terms of true positive (TP), false positive (FP), true negative (TN), and false negative (FN) sets. Figure 9 shows how TP, FP, TN, and FN can be defined in the skin lesion area segmentation scenario.

**(Pixel-wise) accuracy** is the percent of pixels in the prediction image that are labelled correctly and can be defined as:

2 $$\begin{aligned} Accuracy = \frac{TP + TN}{TP + FP + TN + FN} \end{aligned}$$

Although *Accuracy* is easy to calculate and understand, it is not useful when the lesion and background classes are extremely imbalanced, i.e., when a class dominates the image and the other covers only a small portion of the image, which is rather frequent in dermoscopic images.

Better metrics for dealing with the class imbalance issue are:

1. The Dice Similarity Coefficient.
2. The Jaccard Similarity Index (and its threshold variant).
3. The Tversky Index.

**The Dice Similarity Coefficient (Dice)** measures set agreement by calculating the size of the union of two sets divided by the average of their size. In terms of TP, FP, and FN counts, Dice can be written as:

3 $$\begin{aligned} Dice = \frac{TP + TP}{(FP + TP) + (TP + FN)} = \frac{2TP}{2TP + FP + FN} \end{aligned}$$

In the case of image segmentation, a higher Dice coefficient indicates that the result matches the ground truth better than results that produce lower Dice coefficients. The Dice score reflects both size and localization agreement, more in line with perceptual quality compared to pixel-wise accuracy [29].

**The Jaccard Similarity Index (JSI)** measures the overlap of two sets. The Jaccard index is 0 if the two sets are disjoint, i.e., they have no common members, and is 1 if they are identical. Our goal is to get as close to 1 as possible. JSI can be expressed in terms of TP, FP, and FN counts as:

4 $$\begin{aligned} JSI = \frac{TP}{TP + FP + FN} \end{aligned}$$

**The Threshold Jaccard Index** metric is a variant of JSI that is meant to penalize results where the percentage of FP and FN errors is above a certain threshold. For the skin lesion area segmentation task, the Threshold Jaccard Index is computed according to the following rule:

- Threshold Jaccard = 0, if JSI $< 0.65$;
- Threshold Jaccard = JSI, otherwise

where the threshold value equal to 0.65 has been proposed in the ISIC 2018 Challenge. The choice of the Threshold Jaccard index metric in place of JSI is based on the observation that the latter does not accurately reflect the number of images in which automated segmentation fails, or falls outside expert interobserver variability, i.e., JSI is overly optimistic.

**The Tversky Index (TI)** is an asymmetric similarity measure that generalizes the Dice coefficient and the Jaccard index. It is defined as:

5 $$\begin{aligned} TI = \frac{TP}{TP+\alpha FN+\beta FP} \end{aligned}$$

TI has two parameters, $\alpha$ and $\beta$, with $\alpha + \beta = 1$. When, $\alpha = \beta = 0.5$, TI corresponds to the Dice coefficient, while, when $\alpha = \beta = 1$, TI corresponds to the Jaccard index.

By setting a value of $\alpha$ greater than $\beta$, the FN are penalized more. This is very useful in highly imbalanced datasets where the additional level of control over the loss function yields better small scale segmentation than the normal dice coefficient. Moreover, since TI is a small modification of the Dice coefficient, it is very useful for the cases where a finer level of control is needed, such as in medical imaging.

### Test Data

In order to evaluate the performance of our approach, we consider two different publicly available datasets, namely ISIC 2017 and PH2. The choice of ISIC 2017 is due to the availability of a large annotated test set, since more recent versions of the ISIC dataset do not provide direct access to the test set annotations. In our experiments, we use all the 600 dermoscopic JPEG images from the test data folder and the corresponding 600 binary mask images in PNG format from the test ground truth data folder.

In addition to ISIC 2017 data, we use a second dataset of dermoscopic images, called PH2. The PH2 dataset [31] has been realized by the Universidade do Porto, Tecnico Lisboa in collaboration with the Hospital Pedro Hispano in Matosinhos, Portugal. The dataset is composed of 200 RGB dermoscopic images, with a resolution of 768$\times$574 pixels and a magnification of 20$\times$, annotated with ground truth data. The 200 images are divided into benign lesions (80 common and 80 dysplastic nevi) and malignant lesions (40 melanomas). PH2 images are accompanied by ground truth data consisting in binary masks generated via manual segmentation performed by expert dermatologists. Experiments on PH2 are intended to measure the generalization capability of the considered networks on being trained on a dataset A and evaluated on a dataset B, where A and B are from different sources.

### Quantitative Results

We have compared our Attention Squeeze U-Net with other three networks, namely U-Net, Attention U-Net, and Squeeze U-Net. For all the networks, we carried out a training of 100 epochs using the FTL loss function and we considered for comparison the model that obtained the best results on the ISIC 2017 test set. The complete source code for all the four networks, written using Tensorflow 2 and Python 3, is publicly available at: https://github.com/apennisi/att_squeeze_unet

Table 1 shows the segmentation results obtained on the ISIC 2017 test set by using the Dice Similarity Coefficient and the Threshold Jaccard Index as quality metrics. Attention Squeeze U-Net performs slightly better than the other models, achieving a Dice score of 0.9035 and a Threshold Jaccard score of 0.7758 that are better with respect to the winner submission for the ISIC 2017 lesion segmentation task (which achieved a Dice coefficient of 0.849 and an average Jaccard Index of 0.765 [28]).

**Table 1:** The results of the networks on the ISIC 2017 test set (600 images)

**Network**	**Dice**	** Threshold Jaccard**
*U-Net*	0.8965	0.7591
*Attention U-Net*	0.8766	0.7043
*Squeeze U-Net*	0.8987	0.7597
*Attention Squeeze U-Net*	**0.9035**	**0.7758**

The best results are highlighted in bold

The results in Table 1 shows also that by using a combination of the attention mechanism and the reduction of the network parameters (Attention Squeeze U-Net) allows to improve the performance with respect to using the attention block alone (Attention U-Net), the squeeze block alone (Squeeze U-Net), and neither of them (U-Net). It is interesting to note that, the results that we obtained on the ISIC 2017 test set are better with respect to the ones reported in the literature. For example, our conventional U-Net model achieves 0.8965 Dice score, which is much higher than the 0.847 reported as second best result in the ISIC Challenge 2017. This is due to the fact that we used the FTL loss for training the models in Table 1, which gives the networks a better generalization capability.

Table 2 shows the segmentation results on the PH2 dataset. As stated above, the aim of using a second test set that is independent from the training data is to evaluate the generalization capability of the considered models. The analysis of the results indicates that the two models with a smaller size (i.e., Squeeze U-Net and Attention Squeeze U-Net) perform better than the two larger models (i.e., U-Net and Attention U-Net) in terms of both Dice and Threshold Jaccard scores. This is in line with the principle that limiting the model complexity (in terms of the number of parameters) can help the generalization property of the model.

**Table 2:** Segmentation results on the PH2 dataset (200 images)

**Network**	**Dice**	**Threshold Jaccard**
*U-Net*	0.9083	0.7942
*Attention U-Net*	0.8984	0.7879
*Squeeze U-Net*	0.9231	**0.8753**
*Attention Squeeze U-Net*	**0.9301**	0.8533

The best results are highlighted in bold

In order to investigate how our approach is able to generalize the segmentation problem, we performed additional tests on the ISIC 2018 dataset. We point out that our network has not been re-trained on the ISIC 2018 train set, but it has been launched on the testing set of ISIC 2018 [32, 33] to obtain a set of black and white masks. Then, the masks have been submitted to the challenge website to be evaluated by the system. Table 3 shows that our approach obtains a Dice score of 0.67. We compare our results with three other methods, namely *Polar Res-U-Net++ *[19], *DoubleU-Net *[22], and *BCDU-net *[20], which have been trained on ISIC 2018 data. Considering that our method has not been re-trained, it achieves a good score. Moreover, Attention Squeeze U-Net has less training parameters to learn with respect to the methods that achieve better results.

**Table 3:** Segmentation results on the ISIC 2018 dataset (1,000 images)

**Network**	**Dice Score**
*Polar Res-U-Net++ *[19]	**0.92**
*DoubleU-Net *[22]	0.90
*BCDU-net *[20]	0.85
*Attention Squeeze U-Net*	0.67

The best results are highlighted in bold

### Per-lesion Class Results

In this section, we analyze the segmentation results of the four models on the ISIC 2017 test images by separating them according to the lesion type. The expert dermatologists that are in the group of the authors of this paper performed a visual inspection of the 600 test images from the ISIC 2017 dataset and grouped them in seven classes:

- Actinic Keratoses and Intraepithelial Carcinoma (AKIEC): common non-invasive variants of squamous cell carcinomas. They are sometimes seen as precursors that may progress to invasive squamous cell carcinoma.
- Basal Cell Carcinoma (BCC): a common version of epithelial skin cancer that rarely metastasizes, but it grows if it is not treated.
- Benign Keratosis (BKL): contains three subgroups, namely seborrheic keratoses, solar lentigo, and lichen-planus like keratoses (LPLK). These groups may look different, but they are biologically similar.
- Dermatofibroma (DF): a benign skin lesion that is regarded as a benign proliferation or an inflammatory reaction to minimal trauma.
- Melanoma (MEL): a malignant neoplasm that can appear in different variants. Melanomas are usually, but not always, chaotic, and some criteria depend on the site location.
- Melanocytic Nevi (NV): these variants can differ significantly from a dermatoscopic point of view but are usually symmetric in terms of distribution of color and structure.
- Vascular Lesions (VASC): generally categorized by a red or purple color and solid, well-circumscribed structures known as red clods or lacunes.

**Fig. 10:**
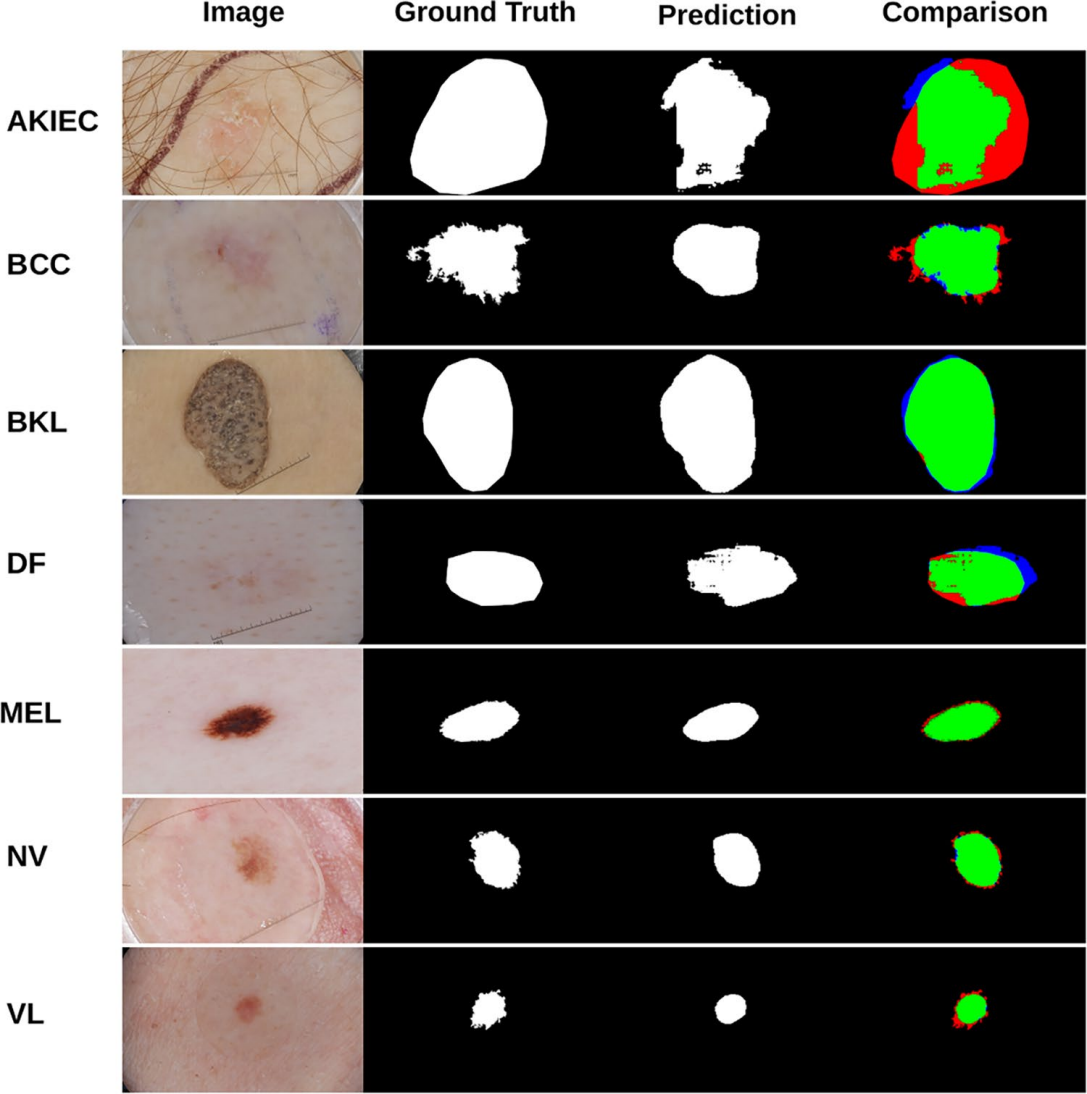
Qualitative results divided per-class on the ISIC 2017 test set obtained by Attention Squeeze U-Net

Figure 10 shows some qualitative segmentation results obtained by Attention Squeeze U-Net divided per-class.

Table 4 shows the quantitative results obtained by all the four considered models. Two clear aspects emerge from the analysis of the results:

1. There is a high inter-class variability for the segmentation results.
2. The four different network architectures produce covariant segmentation results for each lesion class.

**Table 4:** Per-lesion class segmentation results on ISIC 2017 test set

**Lesion Type**	**U-Net**		**Att. U-Net**		**Squeeze U-Net**		**Att. Squeeze U-Net**	
	**Dice**	**Th.Jacc.**	**Dice**	**Th.Jacc.**	**Dice**	**Th.Jacc.**	**Dice**	**Th.Jacc.**
**AKIEC**	**0.7980**	0.3531	0.7036	0.2348	0.7524	0.2756	0.7888	**0.3780**
**BCC**	**0.8775**	**0.7522**	0.8393	0.6227	0.8396	0.6228	0.8531	0.6792
**BKL**	0.8827	0.7372	0.8490	0.6219	0.8356	0.7705	**0.8974**	**0.7847**
**DF**	0.9333	0.8751	0.8829	0.7908	0.9275	0.8652	**0.942**	**0.8909**
**MEL**	0.8839	0.7274	0.8722	0.7155	0.8938	0.7771	**0.9088**	**0.7955**
**NV**	0.9403	0.8754	0.9228	0.8274	0.9389	**0.8979**	**0.9535**	0.8976
**VL**	0.8890	0.7769	0.8426	0.5985	**0.8895**	**0.7871**	0.8482	0.5578

The best results are highlighted in bold

All the considered models obtain good results on benign keratosis (BKL), melanoma (MEL), and melanocytic nevi (NV). Bad results are obtained on Actinic Keratoses and Intraepithelial Carcinoma (AKIEC) by all the models. In particular, the results of our Attention Squeeze U-Net are not optimal on AKIEC, BCC, and VL lesions. However, it is worth noticing that the number of test samples for those categories is limited ($\le$ 30) and that it is considerably fewer than the samples in the other categories. The biological complexity in AKIEC, BCC, and VL is particularly relevant. The clinical examination by tactile sensation, in the absence of any anamnestic information, leads to overestimate some not flat lesions (Breslow thickness > 1 mm) in order to avoid a bad prognosis. For this reason, AKIEC, BCC, and VL samples are less present in public datasets.

### Attention Squeeze U-Net Error Analysis

In this section, we provide a deeper error analysis for our network. Attention Squeeze U-Net generates 47 samples (over 600) of the ISIC 2017 test set where the segmentation can be considered unusable, i.e., the Threshold Jaccard Index is $< 0.65$ [9].

For the AKIEC category, FN errors are mostly related to lesions with low pigmentation and low contrast, while FP errors may be related to three-dimensional lesions, i.e., thick lesions with focus plans at different levels and weak contrast between diseased skin and healthy skin. Failures in BCC category are due to FN errors, which are related to weak contrast and pigmentation regression and to weak contrast between diseased skin and healthy skin. FNs are the majority of the errors for the images classified as MEL that presents a low JSI. Those FN errors are due to pigmentation regression and incomplete acquisitions that occur when dealing with large sized lesions. In NV category, the segmentation failures seem related to FN errors caused by the incomplete acquisition of the lesion and to morphological heterogeneity. FN errors for the samples in the BKL category seem related to images with low contrast, presence of regression, and large sized lesions.

Overall, it should be underlined that in many cases diagnosis based on image alone can be strongly improved by adding specific related information such as anatomical site of the lesion, gender, age, fototype (which could be derived from an image taken from a contro-lateral healthy site) and other anamnestic information. Particularly, follow-up of specific lesions at weeks/months of distance may represent a strong support to further improvement, since this may represent the evaluation of the E feature (evolution) within the ABCDE rule.

### Runtime Performance on Embedded Systems

To demonstrate the capability of the Attention Squeeze U-Net model to run on embedded devices, we carried out runtime performance tests on both an Android phone and an NVIDIA Jetson Nano.

#### Android phone

We used a smartphone equipped with an Exynos 9825 processor, Android operating system, and the open source framework NCNN [27]. To deploy the application on the Exynos 9825 processor, it has been compiled for an ARM architecture AArch64 and a minimum android API:android-24. The final size of the model on Android is about 10 MB and the inference process is completed in about 1.5 s.

#### Nvidia Jetson Nano

Moreover, we carried out a runtime performance test on a Jetson Nano. We measured the execution time of each network on a set of 30 randomly chosen images from the ISIC 2017 test set. As stated above, it is important to note that, we did not optimize the networks using the NVIDIA framework TensorRT.

Table 5 shows the inference time results of all the networks used in this paper. Our network needs an average time of 0.59 s for processing a single image, only 0.02 s more than the standard Squeeze U-Net.

**Table 5:** Execution time experiments using a Jetson Nano on 30 dermoscopic images

**Network**	**Average Execution Time (s)**	**STD**
*U-Net*	1.2	1.55
*Attention U-Net*	1.3	1.63
*Squeeze U-Net*	0.57	1.34
*Attention Squeeze U-Net*	0.59	1.41

Considering that

1. Attention Squeeze U-Net has to learn about 100k parameters more than Squeeze U-Net;
2. The proposed network has better performance;

the obtained results are significant.

## Conclusions

Deep learning based methods have the potential to improve melanoma detection at an early stage by helping in tracking the lesion evolution. Lesion area segmentation is the first step to create an artificial intelligence system that is able to quantitatively compare images of the lesion captured at different time moments. In this work, we have described a lesion area segmentation for dermoscopic images called Attention Squeeze U-Net. Its architecture combines successful ideas from the literature, namely the attention mechanism from Attention U-Net [24], the reduced number of parameters from Squeeze U-Net [23], and the symmetrical shape from U-Net [12].

Attention Squeeze U-Net has a reduced number of parameters, which is compatible with the computational power of embedded devices, and, at the same time, segmentation results comparable with larger models (in terms of the number of trained parameters). Experimental results, conducted on two different publicly available datasets, demonstrate the effectiveness of the proposed model in accurately segmenting dermoscopic images.

We are strongly convinced that the availability of more and more powerful embedded devices (including smartphones) will enable, in the very near future, the patients to run locally the lesion segmentation task, thus preserving their privacy and being proactively involved in the early detection of melanoma.

## Footnotes

1 https://developer.android.com/ndk

2 https://www.lunarg.com/vulkan-sdk/

3 https://developer.nvidia.com/embedded/jetson-nano-developer-kit

4 https://developer.nvidia.com/tensorrt
